# Development of a Nanotechnology Matrix-Based Citronella Oil Insect Repellent to Obtain a Prolonged Effect and Evaluation of the Safety and Efficacy

**DOI:** 10.3390/life13010141

**Published:** 2023-01-04

**Authors:** Celio Takashi Higuchi, Caroline Cianga Sales, Newton Andréo-Filho, Tereza Silva Martins, Helena Onishi Ferraz, Yasmin Rosa Santos, Patricia Santos Lopes, Jeffrey Ernest Grice, Heather Ann Elizabeth Benson, Vania Rodrigues Leite-Silva

**Affiliations:** 1 Instituto de Ciências Ambientais Químicas e Farmacêuticas, Universidade Federal de São Paulo, Diadema CEP 09913-030, SP, Brazil; 2 Therapeutics Research Centre, The University of Queensland Diamantina Institute, Translational Research Institute, Brisbane, QLD 4102, Australia; 3 UniSA Clinical and Health Sciences, University of South Australia, Basil Hetzel Institute for Translational Health Research, Adelaide, SA 5001, Australia; 4 Curtin Medical School, Curtin University, Perth, WA 6845, Australia

**Keywords:** citronella oil, natural products, repellent and nanotechnological matrix, nanostructured lipid carrier (NLC)

## Abstract

Mosquito-borne diseases affect millions of people worldwide each year, and the use of a topically applied insect repellent is an economically viable preventative health practice. The general objective of this work was to encapsulate citronella oil (CO) in a nanostructured lipid carrier (NLC) to formulate a topical repellent with a long duration of efficacy on the skin and a good safety profile based on minimizing skin penetration. In the studied CO, the main chemical constituents of geraniol, citronellal, and citronellol were identified and subsequently used as markers for the in vitro skin permeation testing (IVPT). An optimal NLC encapsulating CO formulation was developed and had an average particle size of 350 nm. The NLC was then formulated in combination with CO at ratios of 2:1, 1:1, and 1:2 CO:NLC-CO as oil-in-water (O/W) emulsions and compared to CO in the same O/W emulsion base (all at 10% CO in the final O/W topical formulation). The markers geraniol, citronellol, and citronellal were detected in all samples tested F1 (10% CO in O/W emulsion) and F3 (10% CO/NLC-CO 1:1 in O/W emulsion). Even the percentages of F3 markers were higher than F1. The recovery of the percentage balance (based on the total remaining on the skin surface, on the skin, and penetrated through the skin to the receptor) of geraniol, citronellol, and citronellal markers for F1 and F3 was 7.70% and 11.96%; 25.51% and 31.89%; and 5.09% and 4.40%, respectively. The nanoparticle lipid solid forms a repellent reservoir on the skin surface, releasing the active ingredients slowly through volatilization, extending the repellent action, and reducing permeation through the skin. It is possible to assume that the remaining 92.30% and 88.03%; 74.49% and 68.11%; and 94.10% and 95.60% of geraniol, citronellol, and citronellal markers of F1 and F3, respectively, were lost to evaporation. In the in vivo efficacy test carried out with the *Aedes aegypti* mosquito, F3 was the optimal formulation, providing the greatest repellent action compared to free oil in O/W emulsion. Thermal analysis showed that the NLC-CO raised the boiling point of the encapsulated CO compared to the free oil, suggesting that the controlled release of the CO was a possible mechanism for its prolonged effect. We concluded that the nanocarriers developed with CO were stable and provided improved mosquito-repellent efficacy with minimal skin penetration of the CO actives over 24 h. Indeed, regardless of whether the CO was applied as free oil, a 1:1 mixture of CO (pure/free oil) or NLC-CO applied in an O/W emulsion can be considered safe for topical application due to minimal skin penetration.

## 1. Introduction

The use of repellents to prevent insect-borne diseases is a recommended cost-effective healthcare practice. The search for improved repellents that are safer, longer lasting, and more effective continues to be at the forefront of research in this area. Vector control and personal protection are very important to minimize the emergence of disease, especially in regions infected with the Zika virus, West Nile virus, Chikungunya virus, yellow fever, dengue, and malaria [1,2,3].

In the personal care market, numerous repellents of synthetic or natural origins are sold, the most common being N, N-diethyl-3-methylbenzamide (DEET), which is effective and has a low cost and has shown excellent repellency against mosquitoes and other biting insects [1]. DEET binds to the olfactory receptors of mosquitoes, inhibiting biting [4,5]. The concentration of DEET in repellent formulations can range from 4 to 100%. Whilst reports of serious adverse reactions to DEET-containing products are rare [6], DEET has been reported to cross the skin barrier, reaching deeper layers of the skin by diffusion and rapidly entering the blood circulation [7]. There have also been reports of the development of insect resistance to DEET after repeated use of the product [8].

Despite the effectiveness of DEET, in recent years, consumers have shown great interest in repellent products containing plant-based ingredients. This has been promoted by claims that natural products are safer and more sustainable, although this is sometimes disputed [8,9,10,11], but also by the more general drive amongst consumers to reduce chemical use and return to nature.

Based on a knowledge of plants with potential insect-repellent action, ethnobotanical studies are being carried out for the discovery and development of new natural products with effective repellent action [8]. Commercial insect repellent products containing plant-based ingredients have gained increasing popularity among consumers, as these are commonly perceived as “safe” in comparison to long-established synthetic repellents [9,12].

Citronella oil (CO) is an important essential oil and one of the most widely used natural repellents, with an applied concentration range of 5 to 10% (p/v), providing protection for up to 2 h [13,14]. CO is derived from two closely related types of grass: *C. winterianus* Jowitt is known as ‘Java-type’ and *C. nardus* (L.) Rendle, known as ‘Ceylon-type.’ These can be distinguished morphologically by the shape and length of their leaves and chemically by the composition of the essential oil obtained from them [15,16,17]. It demonstrates excellent repellency activity because it has a notable presence of monoterpenes, sesquiterpenes, and phenols [9,18] that evaporate when applied to the skin surface, repelling mosquitos [19].

The goal of product development strategies is to increase the repellency time of these natural products by prolonging their residence time on the skin [20]. Encapsulation of CO in a nanostructured lipid carrier (NLC), where the oil can form a reservoir within the lipid matrix, is proposed as a strategy to provide better control of the release of the active components of the oil into the environment. NLC have an oil phase arranged as nanocompartments within a solid lipid [11,21], thereby facilitating the incorporation of the CO. We have developed NLC-based formulations of CO and characterized their physical properties, stability, skin permeation, and duration of mosquito repellency to determine if NLC can improve the safety and efficacy.

## 2. Materials and Methods

### 2.1. Identification of Citronella Oil

CO (d = 0.866 g/mL at 20 °C) was from Mapric^®^ Pharmaceutics Products Ltd. (São Paulo, Brazil), whose analysis report indicated that it came from the species *Cymbopogon nardus* under CAS: 8000-29-1 with a specific CO content of 70% and completed with analytical grade mineral oil. CO was diluted in 99.99% ethyl alcohol (Pharmco-Aaper, São Paulo, Brazil), obtaining a final concentration of 28.84 μg/mL. This procedure was performed in triplicate. All samples were stored at 5 °C before performing the analysis.

The compounds present in the CO were identified by a gas chromatograph coupled to a mass spectrometer (GCMS-QP2010 Plus, Shimadzu Corp, Kyoto, Japan) with an Rtx^®^-5MS column: 30 m, 0.25 mm ID, 0.25 µm (Restek, Bellefonte, PN, USA). The column temperature schedule was 70 °C (2 min) to 100 °C (10 min) at 5 °C/min, 100 to 300 °C at 15 °C/min, and 300 °C (25 min). Injector and detector temperatures were maintained at 250 °C, and the carrier gas was helium at a flow rate of 1 mL/min [19]. The compounds were identified by similarity index analysis using the Wiley Registry^®^ of Mass Spectral Data, 12th edition.

According to the terms of the CGen Resolution No. 27 of 25 August 2021 (Genetic Heritage Management Council, Ministry of the Environment, Brasilia, DF Brazil), the studied oil is not a component of the national genetic heritage. CGen’s main mission is to make the national access and benefit-sharing system a tool for the economic, social, cultural, and environmental development of Brazil, promoting the conservation of biodiversity [22].

### 2.2. Quantification of Citronella Oil

Two stock solutions were prepared: 171.2 μg/mL citronellal (d = 0.856 g/mL at 20 °C) (Sigma-Aldrich Quimica Brasil Ltd., São Paulo, Brazil) and 168.2 μg/mL citronellol (d = 0.841 g/mL at 20 °C) (Labsynth Laboratories Ltd., São Paulo, Brazil) using 99.99% ethyl alcohol as solvent (Pharmco-Aaper, São Paulo, Brazil).

Solutions were prepared by diluting the stock solution with the same solvent to serial concentrations from 1.71 to 17.12 μg/mL for citronellal and from 0.84 to 8.41 µg/mL for citronellol standards. Each sample was prepared in triplicate and injected into the GC-MS under the conditions described. Analytical curves for citronellal and citronellol were prepared based on the peak areas of each solution.

### 2.3. Development of a Nanostructured Lipid Carrier with Citronella Oil (NLC-CO)

Glyceryl monostearate, sodium lauryl sulfate, and CO were purchased from Mapric Ltd. (São Paulo, Brazil), polyvinyl alcohol-medium viscosity was donated by Chemistry DC (Diadema, Brazil), and cetyl trimethyl ammonium chloride was donated by Aqia Química Industrial Ltd. (São Paulo, Brazil). All other reagents were of analytical grade. Table 1 summarizes the components of the oil and aqueous phases of the optimal NLC-CO formulation, including the cationic surfactant (cetyl trimethyl ammonium chloride) [23] generated in the development program:

The NLC-CO was prepared by the high-pressure homogenization method after prior emulsification of the aqueous and oil phases [24]. In brief, the aqueous and oil phases were heated separately to 70 °C with the complete dispersion of the polyvinyl alcohol (aqueous phase) and total melting and mixing of the oil phase. The aqueous phase was added to the oil phase with constant stirring (HSC hot plate stirrer, Velp Scientifica Srl, Usmate, Italy) for 10 min. The hot emulsion was then subjected to high-pressure homogenization (Nano DeBEE, BEE International Inc, MA, USA) under a pressure of 20,000 psi and back pressure of 2000 psi for 10 min (closed system). The NLC-CO was then allowed to cool to room temperature and stored in a Falcon glass bottle for further characterization.

### 2.4. Oil-in-Water Emulsion (O/W Emulsion)

Ceteareth-6 (and) stearyl alcohol (Cremophor^®^ A6), Ceteareth-25 (Cremophor^®^ A25), cetyl alcohol (Lanette^®^ 16), and octyldodecanol (Eutanol^®^) were donated by BASF S.A. (São Paulo, Brazil); cetostearyl alcohol and polysorbate 60 (Polybase^®^ CT) were donated by Aqia Quimica Industrial Ltd. (São Paulo, Brazil). 5-chloro-2-methyl-4-isothiazolin-3-one and 2-methyl-4-isothiazolin-3-one (ProTeg^®^ GC) from Proserv Quimica Ltd. (São Paulo, Brazil) and glycerin from Labsynth Laboratories Ltd. (Diadema, Brazil). All ingredients were analytical grade.

The formulation of the O/W emulsion is stated in Table 2. The oil phase ingredients were melted together at 70 °C and the aqueous phase at 75 °C. The phases were combined and stirred until complete homogenization to prepare the O/W emulsion. ProTeg^®^ GC (preservative) was added at around 40 °C.

### 2.5. O/W Emulsion with Citronella Oil or NLC-CO

The O/W emulsion (Table 2) was used as the base for formulations F1 (with 10% CO), F2 (with 7.3% CO and 36.0% NLC-CO ratio 2:1 [equivalent to CO of 2.7%]), F3 (with 5.0% CO and 66.6% NLC-CO ratio 1:1 [equivalent to CO of 5.0%]), and F4 (with 2.7% CO and 97.3% NLC-CO ratio 1:1 [equivalent to CO of 7.3%]) as shown in Table 3. The amount of CO added up to 10% of the total of all formulations (from F1 to F4). CO or mixtures of CO and NLC-CO (CO/NLC-CO) were added into the O/W emulsion and stirred until complete homogenization.

### 2.6. Thermal Analysis

Differential scanning calorimetry (DSC) and thermogravimetric analysis (TGA) measurements were performed for CO, caprylic capric acid triglyceride (CCT), NLC-CO, and NLC using a simultaneous thermal analyzer DSC/TGA (Discovery SDT 650, TA Instruments, Newcastle, DE, USA). DSC/TGA curves were obtained at a heating rate of 10°C/min in the temperature range from room temperature to 700 °C, under a dynamic N_2_ atmosphere (100 mL/min), using an alumina crucible (90 mL), with ca. 10 mg of the sample mass which was closed by an alumina lid [25,26].

### 2.7. Particle Size Analysis

Particle size analysis was performed by laser diffraction with a Cilas 1190 Particle Size Analyzer (WI, USA). Physical stability monitoring of NLC-CO, O/W emulsion, and F3 at room temperature was performed by analyzing samples on days 0, 1, 7, 14, and 28. A mass of 2.0 g of each sample was weighed after dilution in 50 mL of purified water at 25 °C in triplicate. The volume-weighted diameters d(v) 0.10, d(v) 0.50, d(v) 0.90, and d(v) average were used to characterize the dispersions. Mean values, standard and relative deviations were calculated [27].

### 2.8. Determination of pH

The pH of the NLC, O/W emulsion, and F3 was determined at room temperature on days 0, 1, 7, 14, and 28 using a previously calibrated pH meter (Hanna Instruments Brasil, Brazil). About 2.0 g of each sample was diluted in 50 mL of purified water with pH analyses performed in triplicate, and the mean, standard deviation, and relative standard deviation were determined. The results were submitted for analysis of variance to compare the samples.

### 2.9. Efficacy Test of Mosquito Repellency

The efficacy test was carried out with adult female *Aedes aegypti* mosquitoes aged between 5 and 10 days without blood supply and fasting (free from 10% sucrose solution) for at least 12 h before the study exposure time. The mosquitoes were raised in a vivarium with a controlled temperature of 27 ± 3 °C, a relative humidity of 80 ± 10%, and a photoperiod of 12 h of light and 12 h of darkness [28].

Square glass cages measuring 40 × 40 × 40 cm (64,000 cm^3^), with two screened sides and a front opening to insert the participants’ forearms, were used. Each cage had 55 female mosquitoes in the proportion of one mosquito/1160 cm^3^.

Participants aged 18 to 65 years, male and female, were previously instructed on the study protocol and provided informed consent. This work was approved by ethics committee 8227—Ecolyzer Laboratory (São Paulo, Brazil). An attractiveness test with trial research participants was conducted prior to the application of any repellent products. This procedure is important to verify if the research participants were suitable for the study. Participants’ forearms were exposed to mosquitos. At least 5 mosquito landings should be recorded within a 1 min period. All participants had to pass this test to be admitted to the study [29].

**Figure 1:**
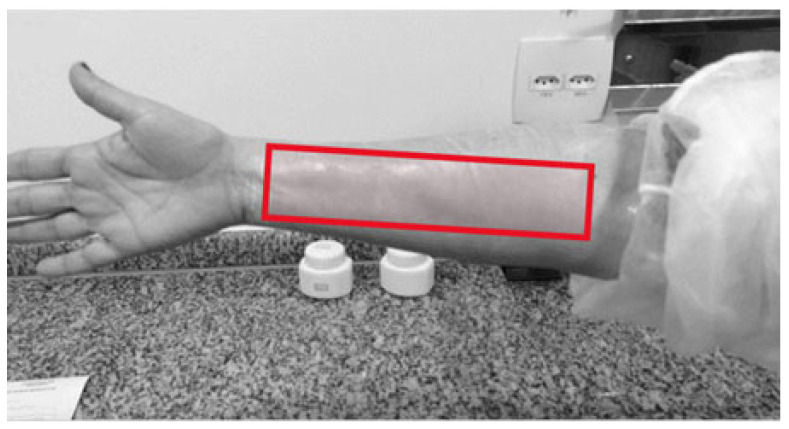
Forearm area for sample application (red marking) (author’s photo).

The sample dose was applied to an equivalent proportional area of the forearm of each volunteer calculated using the formula {[(M1 + M2 + M3 + M4)/4 × C]/600 × 2} where: M1 is the circumference of the forearm at the wrist (cm); M2 and M3 the two equidistant measurements of the circumference of the forearm between the elbow and wrist (cm); M4 the circumference of the forearm at the height of the elbow (cm) and C the length of the forearm (cm). Figure 1 indicates that areas of the forearms were marked ready for application of the test formulation.

Four formulations were evaluated on three participants: F1, F2, F3, and F4. The topical application rate for each formulation was 1 g/600 cm^2^ of skin, applied by rubbing on the arms [28].

The sample-treated forearm was exposed to the mosquitos inside the cage for 5 min at each test time. The first efficacy evaluation was carried out 30 min after the application of the sample, and the following evaluations were at intervals of 1 h until notification of the first mosquito bites. During the interval, the participants had to remain in the laboratory and avoid touching or rubbing the site applied with the sample. All tests were carried out during the day, as *A. aegypti* mosquitoes have a diurnal habit. The repellency time of the sample (100%) was determined by the absence of mosquito bites in the treated research participants. Each test was concluded at the first bite during an evaluation [28].

### 2.10. Safety Test—In Vitro Skin Penetration (IVPT)

#### 2.10.1. Analytical Method

Citronellal, citronellol, and geraniol components of CO were quantified as markers of the skin penetration of active components following topical application of the CO-containing formulations. All quantification was achieved by HPLC on a Shimadzu HPLC system comprising an LC-20AT solvent pumping system, CBM-20A autoinjector, CTO-20A column oven, SPD-20A (DAD) UV diode array detection system and controlled by LC Solution Version 1.25 SP4 software. Chromatographic separation was achieved on an Eclipse XDB-C18 column (150 mm × 4.6 mm), 5 µm with a pre-column C18 (12.5 mm × 4.6 mm) stabilized at 25 °C. The mobile phase was acetonitrile and water 30:70 (*v*/*v*) (one channel) and phosphoric acid (3.2 mL/L of ultrapure water) (second channel) mixed to reach a final pH of 2.1 and flow rate 1.0 mL/min. The sample injection volume was 20 μL.

Analytical curves were constructed using concentrations from 7.2 to 460.5 µg/mL for 95% citronellal (Sigma-Aldrich), 0.52 to 33.0 µg/mL for 98% citronellol (Labsynth), and 0.04 to 2.7 µg/mL for 98% geraniol (Sigma-Aldrich). All analyzes were performed in triplicate. Mean based on peak areas, standard deviation, and relative standard deviation values were determined. O/W emulsion, F1 in immediate time (t = 0 h) and after 45 days (t = 45 days), and shredded skin samples were also analyzed at room temperature (20 a 25 °C).

Sample preparation for analysis: 200.0 mg of O/W emulsion and F1 were weighed into a 50 mL Falcon tube, and 15.0 mL of methanol was added (Avantor, Radnor, PA, USA). Following 60 min in an ultrasonic bath (Solid Steel and 40 KHz), the solutions were filtered using hydrophobic polytetrafluoroethylene (PTFE) with a pore size of 0.22 µm and diluted 1:8 *v*/*v* with methanol. The diluted solutions were injected into the HPLC. This procedure was performed in triplicate.

#### 2.10.2. IVPT Protocol

The IVPT was performed using human skin obtained from plastic surgery as approved by the ethics committee of UNIFESP (2726514). The adipose tissue was removed by dissection, leaving only the epidermis and dermis, which was stored in a freezer (−20 °C). On the day of the experiment, the skin was thawed at room temperature and hydrated with phosphate-buffered saline (PBS) pH 7.4 one hour before initiation of the experiment with Franz Cell. The skin integrity was assessed by transepidermal water loss (TEWL) using a Tewameter^®^ TM 300 Courage + Khazaka Electronic. TEWL values below 10.0 g/m^2^/h^1^ were accepted [30,31].

The receiver compartment contained 2.5 mL of a mixture of water and absolute ethanol 1:1 (*v*/*v*) [19,32,33] as required to provide adequate solubility of CO components [34]. The system was adjusted to 37 °C by immersion in a circulating water bath, providing a membrane surface temperature of 32 ± 2 °C. The diffusion surface area was 3.79 cm^2^.

After equilibrating the entire system for 30 min, the application of formulations F1 (CO) and F3 CO/CLN-CO (1:1) was started. The applied donor solution consisted of a weighed sample (equivalent to 20.0 mg CO), and the donor compartment remained open to mimic topical application to the skin surface and allow evaporation to the environment. At predetermined time intervals (0, 1, 3, 8, and 24 h), an aliquot of 1.0 mL was withdrawn from the receptor phase and immediately replaced with an equal volume of prewarmed receptor solution.

After the final sample (24 h), the system was dismantled and the exposed portion of skin with a surface area of 3.79 cm^2^ was excised using surgical scissors. Two cotton swabs were then applied on the surface of the skin to remove the product residues. The skin surface was exposed to 2.0 mL of methanol in a refrigerator for 24 h, and the resultant solution was filtered using hydrophobic PTFE with a pore size of 0.22 μm prior to HPLC analysis. This assay was performed in quintuplicate (5x).

The dermis and epidermis (DE) were cut into small pieces using sterile scissors. They were immersed in 2.0 mL of methanol in a 2.0 mL Eppendorf tube in a refrigerator (12 to 15 °C) for 24 h. Subsequently, the sample was shaken on a Vortex shaker for 3 min and kept in an ultrasonic bath (SolidSteel and 40 kHz) for 20 min. A volume of 1.5 mL of the solution was collected, filtered using hydrophobic PTFE with a pore size of 0.22 μm, and analyzed by HPLC. The collected receptor solution samples were also analyzed by HPLC.

The areas corresponding to the peaks of each of the citronellal, citronellol, and geraniol markers were recorded, and the mean, standard deviation, and relative standard deviation values were determined.

## 3. Results and Discussion

### 3.1. Identification of Citronella Oil

The compositions of citronella oil (*C. nardus*) (Java type) determined by GC-MS are summarized in Table 4. Citronella oil was found to contain a mixture of several terpenes. The most abundant component was citronellal (71.13%), followed by citronellol (7.10%) [19].

Traditionally, the plant known as citronella has a notable presence of terpene alcohols in the composition of its essential oil, which provide an effective repellency action [29,35,36]. In general, the essential oil extracted from citronella includes more than 80 types of terpenes such as alcohols and aldehydes, including geraniol, citronellal, citronellol, linalool, that are considered the major components with effective insect repellency action comparable to DEET [8,37].

**Figure 2:**
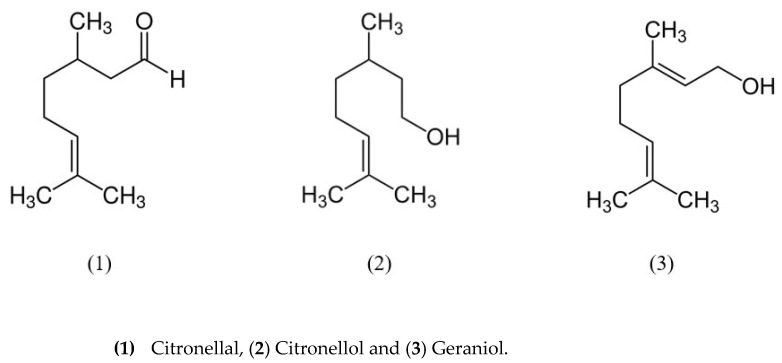
Molecular structures of citronellal (**1**), citronellol (**2**) and geraniol (**3**) markers.

Table 5 shows the general properties of citronellal, citronellol, and geraniol markers in CO and Figure 2 their respective molecular structures:

Commercially, CO is classified into two chemotypes, Ceylon citronella oil obtained from *C. nardus* (L.) Rendle and Java CO obtained from *C. winterianus* Jowitt. The Ceylon chemotype consists of geraniol (18 to 20%), limonene (9 to 11%), methyl isoeugenol (7 to 11%), citronellol (6 to 8%), and citronellal (5 to 15%). The Java chemotype consists of citronellal (32 to 45%), geraniol (11 to 13%), geranyl acetate (3 to 8%) and limonene (1 to 4%), citronellol (%) [40]. In addition, Java CO is considered superior to Ceylon citronella as it makes up about 85% of important compounds such as citronellal, citronellol, and geraniol compared to Ceylon oil, containing only 55 to 65% [41]. It was noted that the main constituents of Ceylon chemotype CO used in the current study were different from those published in the literature [40,41]. It is known that both citronellal and citronellol have proven repellent activity [20,42].

### 3.2. Quantification of Citronella Oil

Citronellal and citronellol contents were 50.10 and 2.4%, respectively, from the analytical curves of the standards [R (0.9920) and R (0.9929) from citronellal and citronellol, respectively]. A *p*-value greater than 0.05 considered this model statistically significant. Citronellal, citronellol, and geraniol markers are considered repellency markers of *Cymbopogon* species and were chosen for the quantification and characterization of the formulations developed in this study.

According to the literature, the species *C. nardus* can vary in terms of its chemical composition from 5.2 to 46.8% for citronellal and 3.0 to 21.8% for citronellol [43]. Ribeiro et al. (2016) found 47.12% of citronellal and 11.07% of citronellol [44]. Baranauskiene et al. (2006) found citronellal 33.9%, citronellol 8.7%, limonene 5.2% and geraniol 16.4% [45].

Citronellal was present in a higher percentage, and citronellol at a lower percentage than in the literature reports. Wide differences in essential oil composition are not uncommon, and divergences in the chemical content of the constituents can be explained in terms of genetic variability, geographic location, harvest time, climatic conditions, cultivation management, age of plant material, and storage period and conditions. Among others [45,46,47].

### 3.3. Thermal Analysis

**Figure 3:**
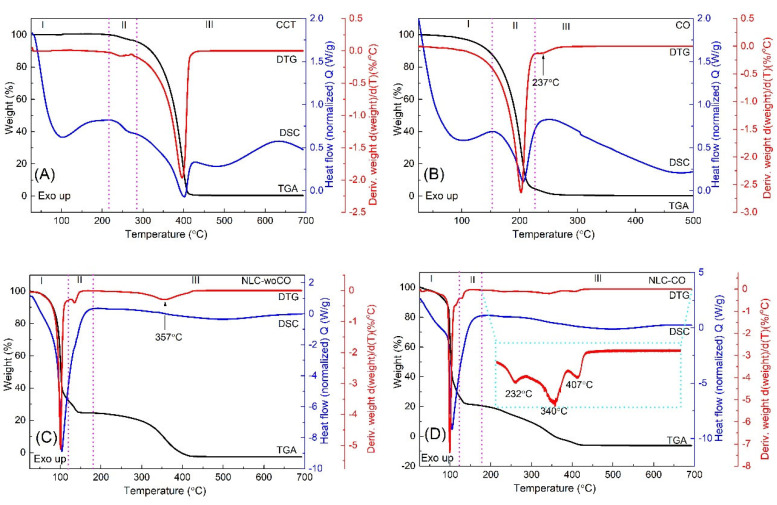
TGA/DTG and DSC curves of (**A**) caprylic acid triglyceride (CAT), (**B**) citronella oil, (**C**) NLC, (**C**) and (**D**) NLC-CO.

The TGA/DTG and DSC curves of (A) caprylic acid triglyceride (CAT), (B) CO, (C) NLC, and (D) NLC-CO are shown in Figure 3, and data extracted from the curves are summarized in Table 6.

The CAT TGA curve (Figure 3A) showed two weight loss events. The first event (from 215 to 285 °C) presented a mass loss of 4.1% and the second event in the temperature range from 285 to 700 °C presented a weight loss of 95.4% with a peak temperature of the DTG in 245 and 396 °C, respectively, corresponding to CAT evaporation, confirmed by the presence of two endothermic events in the DSC curve, observed at 266 and 401 °C, respectively. No weight loss was observed before 215 °C [48,49].

Figure 3B shows the TGA curve for CO. The mass loss started around 50 °C and ended at approximately 290 °C, which corresponded to oil evaporation. Boiling was observed on the DSC curve with a peak temperature of 207 °C [38].

Therefore, the two stages of CO mass reduction refer to the first referring to the elimination of volatile components of the essential oil, divided into two ranges, the first below 100 °C and the second between 100 and 150 °C, and the second, in which the mass reduction occurs between 150 and 210 °C, representing a loss of 10 to 15% of the mass, possibly due to the degradation of the remaining compounds of the oil or the fixed oil, used for a possible dilution.

Analyzing the TGA/DTG and DSC curves of the NLC formulation (NLC and NLC-CO), Figure 3C and D, respectively, there was a weight loss of around 70% in the temperature range from 25 to 120 °C and peak DSC temperature around 100 °C, corresponding to the release of water molecules and BP of water, respectively. This is in accord with the nominal amount of water used for the preparation of NLC and NLC-CO. In this temperature range, a greater weight loss was observed for the NLC NLC-CO, which can be attributed to the beginning of the elimination of CO, as observed for the pure oil (Table 6).

When comparing the TGA/DTG curves of the NLC samples (NLC and NLC-CO) in the temperature range from 180 to 700 °C, the NLC-CO has a different thermal behavior compared to the sample without the oil (NLC). Degradation/evaporation occurs in three mass losses (Table 6), with temperature peaks at 232, 340, and 407 °C, as seen in the DTG curve highlighted in Figure 3D. Considering that the peak at 232 °C is due to the evaporation of CO, we can consider that the NLC provided greater thermal stability to the CO. This information suggests that CO in contact with the human body will volatilize more slowly and consequently, its skin protection time will increase.

### 3.4. Particle Size Analysis

**Figure 4:**
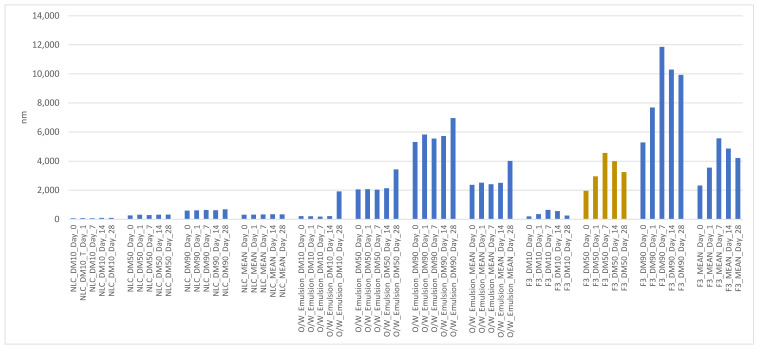
Mean particle size of NLC, O/W emulsion, and F3 monitored for 28 days at room temperature.

**Figure 5:**
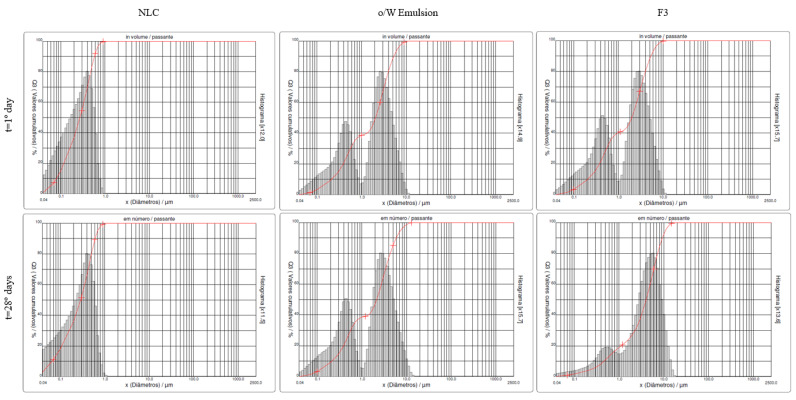
Histograms representing particle size analysis by laser diffraction day 1 and 28 distribution profiles of NLC, O/W emulsion, and F3 at room temperature.

The physical stability of the samples NLC, O/W emulsion, and F3 at room temperature, monitored for 28 days, is shown in Figure 4 and Figure 5:

There was no statistically significant difference in mean particle size (mean diameter: DM) between the NLC, O/W emulsion, and F3 (10% CO/NLC-CO 1:1 in O/W emulsion) (repeated measures Anova, intragroup comparison with 5% significance, post hoc: Bonferroni) monitored for 28 days, except for the comparison of F3_DM_Day_0 to F3_DM_Day_28.

The essential components for the formulation of the NLC were the composition of the cationic surfactant cetyl trimethyl ammonium chloride, glyceryl monostearate, and the lipid content used in the oil phase; and the production parameters utilizing a closed system with high-pressure homogenization technique and an established time of adequate processing [23]. Hot homogenization under high pressure, and the melting of liquid and solid lipids at all stages of production, allowed greater incorporation of the active material into the internal phase of the NLC [50].

The final size obtained may depend on various factors, such as the chemical structure of the lipids, the surfactants used, as well as their chemical interaction. Surfactants function to stabilize the dispersion and the surface of the particles by reducing the interfacial tension between the hydrophobic surface of the lipid core and the aqueous medium, favoring the stabilization of the structures of the nanoparticles. The type of surfactant can influence the ability to incorporate the bioactive of interest and particle formation [27,51,52].

The cationic surfactant was chosen because it has a zeta potential in the range of -40 mV, and the particles are, therefore, less likely to aggregate [23]. The ideal zeta potential value is ?30?mV, which has a greater repulsion and, thus, greater stability compared to smaller values that cause formulation flocculation or instability over time [53]. It was possible to visualize the discrete particle size amplitude of the tested formulation, comfirming the efficiency of the formulation and the process used to produce the monomodal formulations with nanometric dimensions [54]. The surfactants need to be able to stabilize fast the formed droplets when they leave the homogenization gap to avoid subsequent coalescence [27].

Relative to the mean particle size in the O/W emulsion, a distribution profile was observed in a lower percentage of the number of particles from 0 to 1000 nm and a higher percentage from 1000 to 10,000 nm both on day 1 and 28, resulting in a bimodal emulsion.

Polydispersity refers to the breadth and shape of the distribution. When emulsions of the same internal phase content but of very different sizes are mixed, the resulting distribution shows two peaks; this is called a bimodal emulsion. If these two peaks are sufficiently separated, a considerable reduction in viscosity can be obtained due to the small drops that fill the space left by the large drops. This can result in the Ostwald Ripening phenomenon, whereby mass transfer occurs from the smaller to the larger drops [55].

Relative to the mean particle size in F3 (10% CO/NLC-CO 1:1 in O/W emulsion), the incorporation of the oil caused an increase in the size of the nanoparticles (first peak indicated on day 28) and in the average size of the emulsion (the second peak indicated on the day 28) compared to the formulations without the incorporation of the oil (emulsion O/W) [56]. With the stabilization of the emulsifying system and after the days, the oil tended to migrate from the internal phase of the NLC to the external phase of the system, resulting in an increase in the particle size of the incorporated active [31].

### 3.5. Determination of pH

Samples of NLC, O/W emulsion, and F3 were stored at room temperature, and their pH values were determined on days 1, 7, 14, and 28. All samples showed pH values between 3.80 and 4.95 (Table 7):

The NLC presented a gradual reduction in the pH in function. It can be argued that the acid buffering capacity is low. This is in line with what was expected due to the nature of the surfactant since the cationic portion exposed on the surface of the particles would not allow the capture of protons released from the possibly formed stearic acid. On the contrary, they would favor the capture of OH^-^ ions, contributing to the acidification of the environment [23].

The emulsion and F3 were weakly acidic. This pH range is suitable for application to the skin as it is not expected to cause irritation, as it can occur with very basic or acidic products with pH values that are very different from skin pH of about 5.5 [57].

### 3.6. Efficacy test

The protection times for the formulations (F1 and different proportions of the free and nanoencapsulated forms (F2 and F3) are summarized in Table 8.

F1 resulted in an average repellency period of 0.3 ± 0.5 h. The incorporation of CO with NLC at a ratio of 2:1 (F2) resulted in no repellency effect. However, when the ratio was changed to increase the proportion of the NLC component (F3), there was a marked increase in the mosquito repellency period to 4.0 ± 0.0 h. According to Songkro et al. (2012), there is no linear correlation between the release rates and the repellency times of formulations in the CO and encapsulated form [19].

The proportion of 5.0% CO and 5.0% CO included in NLC (F2) was fundamental for extending mosquito repellency. This demonstrates that the higher proportion of CO in the NLC provided a prolonged release of CO-active components. As those volatile components are released and evaporate into the environment, they continue to repel mosquitos from the skin surface. This allowed the effective repellency to continue over the longer period in which they were released by the NLC compared to the more immediate availability of the components from the oil.

### 3.7. Safety Test—Skin Penetration

#### 3.7.1. Analytical Method

**Figure 6:**
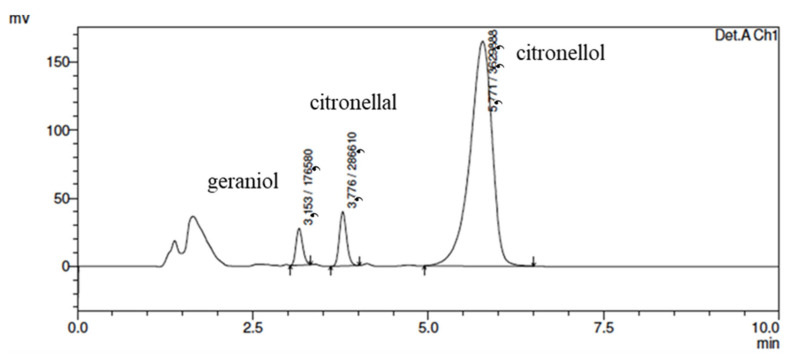
Chromatogram of geraniol, citronellol, and citronellal standards diluted in methanol by HPLC (acetonitrile mobile phase: water (30:70); column C18; a flow of 1 mL/min; injection volume 20 µL; detection 210 nm and run of 10 min).

The HPLC analytical method presented a total running time of 10 min, with elution of geraniol, citronellal, and citronellol at 3.15, 3.77, and 5.77 min, respectively, as shown in Figure 6:

The peak area versus concentration calibration curves was linear for all CO markers (R > 0.999), and all sample preparation methods provided suitable quantification. Samples of citronellal, citronellol, and geraniol markers were analyzed at concentrations of 122.78 (73.69%), 8.79 (5.28%), and 0.716 μg/mL (0.43%), respectively, corresponding to a citronella oil concentration of 166.62 μg/mL in methanol.

In the stability test of O/W emulsion, the citronellal from citronella oil at t = 0 h and 45 days at room temperature (20 a 25 °C) decreased from 98.63 ± 0.02% to 79.78 ± 0.00%. Citronellal has a faster evaporation rate than citronellol and geraniol [58] which may contribute to the change. In contrast, the citronellol and geraniol increased from 98.63 ± 0.00% to 106.00 ± 3.00 and from 72.62 ± 0.06 to 82.40 ± 0.00, respectively over the 45-day period. Citronellal, citronellol, and geraniol are molecules formed from a catalytic reaction of citral. A catalytic hydrogenation reaction for converting from geraniol and citronellal to citronellol is possible. This interconversion could justify the increase in citronellol and geraniol [53].

#### 3.7.2. IVPT Protocol Test

**Figure 7:**
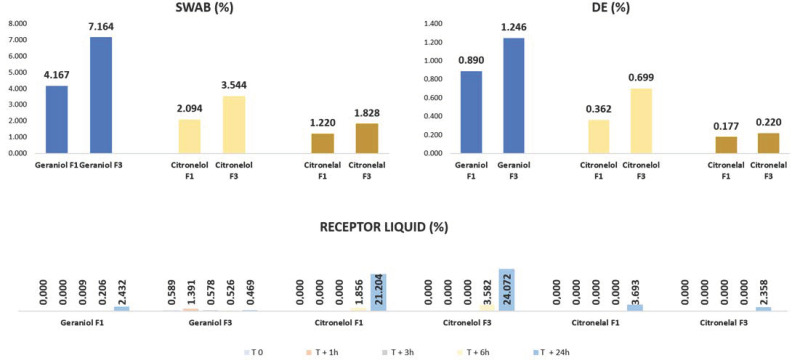
Amount (%) of geraniol, citronellol, and citronellal markers found after application of formulations F1 and F3 on the skin surface (Swab), dermis/epidermis (DE), and receptor liquid.

Figure 7 shows the amount (%) of geraniol, citronellol, and citronellal markers found on the human skin surface after the application of formulations F1 (10% citronella oil in O/W emulsion) and F3 [F3 CO/NLC-CO (1:1)]. The applied donor solution per cm^2^ of skin surface consisted of a weighed sample (equivalent to 20.0 mg CO and 22.69, 278.63 and 3888.65 µg/cm^2^ of geraniol, citronellol and citronellal, respectively).

There was a statistically significant difference amount (%) found in IVPT after 24 h of application of F1 and F3 (mean ± SD, n = 5 and *p* < 0.05—paired *t*-test) between geraniol, citronellol, and citronellal markers compared to the F1 and F3 pairs on the skin surface (Swab) and DE, except with citronellal marker in F1 and F3 in skin surface (Swab).

The nanoparticle lipid solid forms a repellent reservoir on the skin surface, releasing the active ingredients slowly through volatilization, extending the repellent action and, consequently, reducing permeation through the skin [59,60]. However, there was a greater presence of F3 markers in DE. It is important to remember that repellent formulations must have topical action with retention of the active ingredients at the surface layer of the skin (the stratum corneum) to exhibit their insect-repellent action [61].

The amount of CO markers detected in the receptor samples over 24 h following the topical administration of F1 and F3 is important as a determinant of safety as there is no requirement for skin penetration to achieve the desired repellency effect. Overall, there was no statistically significant difference in the amount (%) of geraniol, citronellol, and citronellal markers in the receptor liquid at 0, 1, 3, 6, and 24 h following the application of F1 and F3 (mean ± SD, n = 5 and *p* < 0.05—paired *t*-test). The only exception was the geraniol marker, which was significantly higher in the F1 receptor sample at 24 h than in F3.

The markers geraniol, citronellol, and citronellal were detected in all samples tested F1 (10% CO in O/W emulsion) and F3 (10% CO/NLC-CO 1:1 in O/W emulsion). Even the percentages of F3 markers were higher than F1. The mass balance recovery (based on the total remaining on the skin surface, on the skin, and penetrated through the skin to the receptor) of geraniol, citronellol, and citronellal markers for F1 and F3 was 7.70% and 11.96%; 25.51% and 31.89%; and 5.09% and 4.40%, respectively. We assume that the remaining 92.30% and 88.03%; 74.49% and 68.11%; and 94.10% and 95.60% of geraniol, citronellol, and citronellal markers of F1 and F3, respectively, were lost to evaporation.

It was noted in the first 3 h of the test that citronellol and citronellal markers were not detected in the recipient liquid in both formulations F1 and F3. It is known that citronellal has a higher volatilization rate than the other two markers [58] and is likely to have evaporated rapidly rather than being absorbed into or remaining at the skin surface. In addition, it was noted that the citronellol marker had a higher percentage in the receptor fluid from 3 h onwards in both F1 and F3 compared to the other markers. It is possible to suggest that this occurred due to the physicochemical property of citronellol, in which it presents a higher log P and may have been absorbed into the lipid-rich stratum corneum but slowly permeated deeper into the skin tissues before partitioning into the receptor solution.

Therefore, regardless of whether the oil is free (F1) or encapsulated (F3), skin penetration of the active markers was minimal, so it is possible to suggest that both formulations are considered safe [62].

## 4. Conclusions

The nanostructured lipid carrier developed with this CO (NLC-CO) was shown to be stable and within the expected particle size and safety profile for topical application. The nanoparticle lipid solid forms a repellent reservoir in the skin surface, releasing the active ingredients slowly through volatilization, extending the repellent action and, consequently, reducing permeation through the skin. The mixture of CO (pure/free oil) and encapsulated CO (NLC-CO) in the ratio of 1:1 presented the best mosquito-repellent effect when compared to free oil in O/W emulsion. Thermal analysis performed with DSC and TGA showed that NLC-CO had a higher boiling point than citronella oil, thus suggesting that the prolonged repellent effect was due to slower volatilization of CO on the skin. Over 28 days, the NLC was physically stable and remained within an average nanometer size range. We concluded that the nanocarriers developed with CO were stable and provided improved mosquito-repellent efficacy. It is possible to suggest that regardless of if free oil or a 1:1 mixture of CO (pure/free oil) and NLC-CO is applied in an oil-in-water (O/W) emulsion, both of them are considered safe for topical application.

## Figures and Tables

**Table 1:** Optimal NLC formulation for incorporating CO (NLC-CO).

Ingredients	Phase	(% *w*/*w*) ^1^
Citronella oil (CO)	Oily	7.5
Glyceryl monostearate		7.5
Caprylic capric acid triglyceride		7.5
Cetyl trimethyl ammonium chloride 50%		6.0
Polyvinyl alcohol	Aqueous	1.0
Purified water up to		100.0

^1^*w*/*w* = weight/weight.

**Table 2:** Formulation of O/W emulsion.

INCI Name	Phase	Commercial Name	(% *w*/*w*) ^1^
Ceteareth-6 (and) stearyl alcohol	Oily	Cremophor^®^ A6	3.0
Ceteareth-25		Cremophor^®^ A25	3.0
Cetyl alcohol		Lanette^®^ 16	2.0
Cetostearyl alcohol and polysorbate 60		Polybase^®^ CT	6.0
Octyldodecanol		Eutanol^®^	5.0
Glycerin	Aqueous	Glycerin	5.0
5-chloro-2-methyl-4-isothiazolin-3-one and 2-methyl-4-isothiazolin-3-one		ProTeg^®^ GC	0.5
Purified water up to		Purified water	100.0

^1^*w*/*w* = weight/weight.

**Table 3:** Formulations of O/W emulsion containing citronella oil (CO) or a mixture of CO and NLC-CO (CO/NLC-CO).

Ingredients (% *w*/*w*) ^1^	F1 CO	F2 CO/NLC-CO (2:1)	F3 CO/NLC-CO (1:1)	F4 CO/NLC-CO (1:2)
Citronella oil	10.0	7.3	5.0	2.7
NLC-CO	-	36.0 (2.7 CO)	66.6 (5.0 CO)	97.3 (7.3 CO)
O/W Emulsion up to	100.0	100.0	100.0	-

^1^*w*/*w* = weight/weight.

**Table 4:** Identification of citronella oil components by GC-MS technique.

t_R_ (min) ^a^	Chemical Compounds (INCI) ^b^	% Area
5.609	β-pinene	0.43
6.874	Eucalyptol	0.90
8.607	Linalool	0.47
10.226	p-Menth-8-en-3-ol	5.24
10.535	Citronellal	71.13
10.708	p-Menth-8-en-3-ol	3.48
11.171	Cyclohexanol	0.31
13.996	Citronellol	7.10
20.207	p-Menthane-3,8-diol	0.88
20.797	Citronellol acetate	1.28
22.323	β-Caryophyllene	0.92
24.811	Diethyl Phthalate	0.27
27.523	(R)-(+)-Citronellal	1.18
27.828	Naphthalene	3.56
27.992	Cyclohexane	0.30
28.158	β-Citronellal	2.31
28.932	Farnesyl acetone	0.25

^a^ Time retention and ^b^ Wiley 275. L Mass Spectra Database were used to identify compounds. INCI: International Nomenclature of Cosmetic Ingredients.

**Table 5:** General properties of citronellal, citronellol and geraniol markers in citronella oil [38,39].

Markers	Formula and Molecular Weight (g/mol^)^	d (g/cm^3^) to 25 °C.	Log Pow	SP (mmHg)	MP e BP (°C)
Citronellal	C_10_H_18_O e 154.24	0.855	3.53	2.5 × 10^−1^	< −16 e 207
Citronellol	C_10_H_20_O e 156.26	0.855	3.91	2.0 × 10^−2^	< −20 e < 225
Geraniol	C_10_H_18_O e 154.25	0.890	3.56	3.0 × 10^−2^	< −15 e 229

Legend: d (density), SP (steam pressure), MP (melting point), and BP (boiling point).

**Table 6:** Thermal analysis results (TGA/DTG and DSC) of caprylic acid triglyceride (CAT), citronella oil, NLC, and NLC-CO.

Samples	First Step (I) * 25–215 °C ^♣^ 25–125 °C ^♦^ 25–120 °C					Second Step (II) * 215–285 °C ^♣^ 125–228 °C ^♦^ 120–180 °C					Third Step (III) * 285–700 °C ^♣^ 228–500 °C ^♦^ 180–700 °C ^♠^ 180–275, ^†^ 275–375, ^∙^ 375–700 °C				
	Δw (%)	T_onset_ (°C)	T_peak_ DTG (°C)	T_peak_ DSC (°C)	ΔH (J g^−1^)	Δw (%)	T_onset_ (°C)	T_peak_ DTG (°C)	T_peak_ DSC (°C)	ΔH (J g^−1^)	Δw (%)	T_onset_ (°C)	T_peak_ DTG (°C)	T_peak_ DSC (°C)	ΔH (J g^−1^)
* CAT	-	-	-	101	129.2	4.1	248	245	266	5.2	95.4	365	396	401	77.7
^♣^ Citronella oil	5.3	97	-	95	76.6	90.4	182	201	207	111.2	4.0	236	242	-	-
^♦^ NLC	67.0	93	101	104	1856.1	7.8	133	135	-	-	25.3	313	358	-	-
^♦^ NLC-CO	72.0	96	100	105	1902	7.2	125	130	-	-	^♠^ 8.3 ^†^ 14.1 ^∙^ 4.4	^♠^ 213 ^†^ 315 ^∙^ 405	^♠^ 232 ^†^ 340 ^∙^ 407	- - -	- - -

T = temperature, w = Mass loss, ΔH = Enthalpy (normalized), s = n, * CAT, ^♣^ Citronella oil, ^♦^ NLC and ^♦^ NLC-CO (^♠^ 180–275, ^†^ 275–375, ^∙^ 375–700 °C).

**Table 7:** Determination of pH of samples on days 1, 7, 14, and 28. All data are results obtained from three analyzes (n = 3) (*p* < 0.05).

	pH (SD)			
Samples	Day 1	Day 7	Day 14	Day 28
NLC	3.80 (0.00)	3.79 (0.00)	3.82 (0.00)	3.89 (0.02)
Emulsion	4.75 (0.00)	4.95 (0.00)	4.88 (0.00)	4.95 (0.00)
F3	3.95 (0.01)	3.90 (0.00)	3.93 (0.00)	4.02 (0.02)

Legend: Standard deviation (SD).

**Table 8:** Protection times against *A. aegypti* of formulations (F1, F2, F3, and F4) (*p* < 0.05).

Formulations	Average Protection Time (h) (Mean ± SD, N = 6)	Protection Time of Each Volunteer					
		1	2	3	4	5	6
F1—10.0% CO	0.3 ± 0.5 (6)	1 h	0	1 h	0	0	0
F2—CO/CO:NLC (2:1)	0.0 ± 0.0 (6)	0	0	0	0	0	0
F3—CO/CO:NLC (1:1)	4.0 ± 0.0 (6)	4 h	4 h	4 h	4 h	4 h	4 h

## Data Availability

All the data are available upon request from the corresponding author.
